# Candidate Phyla Radiation (CPR) bacteria from hyperalkaline ecosystems provide novel insight into their symbiotic lifestyle and ecological implications

**DOI:** 10.1186/s40168-025-02077-y

**Published:** 2025-04-07

**Authors:** Yu He, Shiyan Zhuo, Meng Li, Jie Pan, Yongguang Jiang, Yidan Hu, Robert A. Sanford, Qin Lin, Weimin Sun, Na Wei, Shuming Peng, Zhou Jiang, Shuyi Li, Yongzhe Li, Yiran Dong, Liang Shi

**Affiliations:** 1 School of Environmental Studies, China University of Geosciences (Wuhan), Wuhan, China; 2 Archaeal Biology Centre, Synthetic Biology Research Center, Shenzhen Key Laboratory of Marine Microbiome Engineering, Key Laboratory of Marine Microbiome Engineering of Guangdong Higher Education Institutes, Institute for Advanced Study, Shenzhen University, Shenzhen, China; 3 Department of Earth Science & Environmental Change, University of Illinois Urbana-Champaign, Champaign, USA; 4 Shanghai Biozeron Biological Technology Co. Ltd., Shanghai, China; 5 Guangdong Institute of Eco-Environmental and Soil Science, Guangzhou, China; 6 Department of Civil and Environmental Engineering, University of Illinois Urbana-Champaign, Champaign, USA; 7 Institute of Ecological Environment, Chengdu University of Technology, Chengdu, China; 8 Central and South China Municipal Engineering Design and Research Institute Co, Ltd., Wuhan, China; 9 State Key Laboratory of Biogeology and Environmental Geology, China University of Geosciences (Wuhan), Wuhan, China; 10 State Environmental Protection Key Laboratory of Source Apportionment and Control of Aquatic Pollution, Ministry of Ecology and Environment, Beijing, China; 11 Hubei Key Laboratory of Yangtze Catchment Environmental Aquatic Science, Wuhan, China

**Keywords:** Candidate phylum radiation (CPR), Folate, Essential cofactors, Mutualism, Dihydrofolate reductase

## Abstract

**Background:**

Candidate Phyla Radiation (CPR) represents a unique superphylum characterized by ultra-small cell size and symbiotic lifestyle. Although CPR bacteria have been identified in varied environments, their broader distribution, associations with hosts, and ecological roles remain largely unexplored. To address these knowledge gaps, a serpentinite-like environment was selected as a simplified model system to investigate the CPR communities in hyperalkaline environments and their association with hosts in extreme conditions. Additionally, the enzymatic activity, global distribution, and evolution of the CPR-derived genes encoding essential metabolites (e.g., folate or vitamin B_9_) were analyzed and assessed.

**Results:**

In the highly alkaline serpentinite-like ecosystem (pH = 10.9–12.4), metagenomic analyses of the water and sediment samples revealed that CPR bacteria constituted 1.93–34.8% of the microbial communities. Metabolic reconstruction of 12 high-quality CPR metagenome-assembled genomes (MAGs) affiliated to the novel taxa from orders UBA6257, UBA9973, and *Paceibacterales* suggests that these bacteria lack the complete biosynthetic pathways for amino acids, lipids, and nucleotides. Notably, the CPR bacteria commonly harbored the genes associated with essential folate cofactor biosynthesis and metabolism, including dihydrofolate reductase (*folA*), serine hydroxymethyltransferase (*glyA*), and methylenetetrahydrofolate reductase (*folD*). Additionally, two presumed auxotrophic hosts, incapable of forming tetrahydrofolate (THF) due to the absence of *folA*, were identified as potential hosts for some CPR bacteria harboring *folA* genes. The functionality of these CPR-derived *folA* genes was experimentally verified by heterologous expression in the *folA*-deletion mutant *Escherichia coli* MG1655 Δ*folA*. Further assessment of the available CPR genomes (*n* = 4,581) revealed that the genes encoding the proteins for the synthesis of bioactive folate derivatives (e.g., *folA*, *glyA*, and/or *folD* genes) were present in 90.8% of the genomes examined. It suggests potential widespread metabolic complementarity in folate biosynthesis between CPR and their hosts.

**Conclusions:**

This finding deepens our understanding of the mechanisms of CPR-host symbiosis, providing novel insight into essential cofactor-dependent mutualistic CPR-host interactions. Our observations suggest that CPR bacteria may contribute to auxotrophic organisms and indirectly influence biogeochemical processes.

Video Abstract

**Supplementary Information:**

The online version contains supplementary material available at 10.1186/s40168-025-02077-y.

## Introduction

Candidate Phyla Radiation (CPR), also known as *Candidatus* Patescibacteria, represents a large monophyletic radiation within the bacterial domain [1, 2]. The discovery of CPR has significantly expanded the tree of life [3, 4]. With more than 70 different phyla, they account for over a quarter of the phylum-level lineages based on the genome-based phylogenetic analyses by Parks et al. (2017) [5]. CPR bacteria are widespread across diverse ecosystems (e.g., dental plaque, soil, groundwater, hypersaline lakes, wastewater treatment plants, acid mine drainage, and marine sediments) [6–11]. Characterized by streamlined genomes and restricted metabolic capacities, they possess incomplete biosynthetic pathways and lack the full machinery for cellular motility and chemotaxis [12, 13]. Thus, CPR species typically rely on symbiotic hosts for essential nutrients and cellular components.

Episymbiotic associations between CPR bacteria and their hosts have been suggested based on metagenomic and culture-dependent analyses [14–16]. *Nanosynbacter lyticus* TM7x (TM7x), isolated from the human oral cavity, was the first cultivated CPR species. It forms symbiotic relationship with the actinobacterial host *Actinomyces odontolyticus* XH001 (XH001) [17]. In the TM7x-XH001 system, TM7x exhibited a predatory mode and grew at the expense of XH001 [14, 18]. Under prolonged starvation, XH001 cells lost viability and showed severe cell disruption in the presence of TM7x [17]. The parasitic nature of certain CPR members was further supported by the *Absconditabacterales* taxa (e.g., *Ca*. Vampirococcus lugosii and *Ca*. Absconditicoccus praedator) in hypersaline lakes (Salada de Chiprana, Spain and Hotontyn Nur, Mongolia). These taxa possessed the genes encoding virulence factors, including hemolysin and hemolysin translocator, which can disrupt the cell walls of their hosts. Based on these CPR-host interactions, a parasitic lifestyle for CPR members was initially proposed [17, 19, 20].

However, increasing evidence suggests that CPR-host interactions may be more than parasitism. For example, TM7x’s arginine deiminase system may benefit its host XH001 to withstand acid stress and adapt to low pH environments [21]. Additionally, TM7x can enhance biofilm formation, promoting the persistence of XH001 in the oral cavity. It can also prevent XH001 from phage adsorption by downregulating the biogenesis of polysaccharides and phage receptors in the host’s cell wall [22, 23]. Potential mutualistic symbiosis has also been observed in other CPR-containing systems. In the bioreactor for methanogenic benzene degradation, *Ca*. Nealsonbacteria may provide acetate or acetyl-CoA to support growth of the obligate acetoclastic methanogen *Methanothrix*, which benefits both organisms [11]. In groundwater, CPR bacteria constituted up to 40% of the microbial communities, and co-occurrence analysis showed that they significantly enhanced the network connectivity and complexity of these microbial communities [7, 24, 25]. Moreover, *Ca*. Roizmanbacterium ADI133 might donate lactate to its host *Thermodesulfovibrionia*, indirectly influencing biogeochemical cycling in these complex groundwater environments [25]. These studies provide valuable insights into the potential mutualistic interactions between CPR and their hosts, as well as their roles in regulating biogeochemical cycles across various ecosystems.

Despite these advances, understanding of CPR bacteria in their diversity, distribution, interactions with hosts, and ecological significance has remained limited. For example, the available studies on CPR bacteria have been mainly focused on circumneutral and acidic environments. Moreover, the mechanisms underlying CPR-host interactions have been corroborated only in a few culturable systems. To address these knowledge gaps, the CPR diversity, distribution, and association with potential hosts were systematically investigated in an ultrabasic serpentinite-like environmental system located in Sichuan, Southwest China. Through an integrated approach combining geochemistry, metagenomics, and molecular biology, this study provides insights into the unique CPR communities in basic to ultrabasic environments, their potential association with host organisms dependent on essential cofactors, and the ecological significance of such CPR-host interactions.

## Materials and methods

### Sample collection

The sampling sites were located adjacent to a slag storage plant in Panzhihua, Sichuan Province, Southwest China (E 101.60197, N 26.60695). The strongly alkaline leachate (pH > 12) was formed from the reactions between water from various sources (e.g., precipitation, groundwater, and/or the water from a nearby river) and the disposed industrial wastes (e.g., ferrous slags and iron ores), which resembled serpentinization due to hydration and alteration of ultramafic rocks (e.g., olivine and pyroxene) [26–28]. The ultrabasic fluid flowed along the hillslope and was collected in a downstream pond. Nine samples were collected along the runoff of the ultrabasic ferrous slag leachate, including 5 leachate samples (P1-P5) and 4 sediment samples (S1-S4) (Table S1 and references therein). Detailed information on sample collection and DNA sequencing followed the methods described in our recent studies [27, 28].

### Metagenomic assembly and genome binning

Metagenomic reads were quality-trimmed using Trimmomatic v0.39 to discard the Nextera adapters and the low-quality sequences with Phred score below 20 in a sliding window of 4 bp, as well as reads shorter than 50 bp [29]. To explore the taxonomic composition of the nine metagenomes, putative 16S rRNA gene fragments were identified and extracted from the quality-trimmed reads using the phyloFLASH v3.4.1 pipeline [30]. The extracted 16S rRNA genes were taxonomically assigned to the nearest taxonomic unit based on the SILVA SSU rRNA reference database (v138) [31].

Quality-controlled reads from each metagenome and sample type were individually assembled using Spades v3.15.3 with “–meta” mode and k-mer lengths of 21, 33, 55, 77, 99, and 127 nucleotides, resulting in nine individual assemblies and two co-assemblies [32]. Metagenome-assembled genomes (MAGs) were recovered from each assembly using the binning module (parameters: –maxbin2 –metabat1 –metabat2) in the metaWRAP v1.2.1 pipeline [33]. All obtained MAG sets were then consolidated using Das Tool v1.1.4 with a search engine of blast and a score threshold of 0.1 to obtain optimized MAGs [34]. Contamination in the MAGs was removed using MAGpurify v2.1.2 [35]. Briefly, MAGs were profiled based on the four individual modules (phylo-markers, clade-markers, tetra-freq, and GC-content), and the contigs that were outliers in these modules were removed.

The taxonomy of the MAGs was assigned using the Genome Taxonomy Database (GTDB, Release 220) with GTDB-TK v2.4.0 [36]. The quality of MAGs was assessed using CheckM2 v1.0.1, which employs tailored marker sets, refined algorithms, improved handling of fragmented genomes, and enhanced phylogenetic placement to accurately predict genome quality, especially for MAGs from novel lineages with reduced genome size (e.g., CPR superphylum) [37]. Only medium- to high-quality MAGs with estimated completeness > 50% and contamination < 10% were retained and subsequently dereplicated at 95% ANI using dRep v3.2.2 to obtain species-level MAGs for downstream analyses [5, 38].

### Genome annotation

Genomic properties, including the numbers of genes, GC content, and scaffold parameters, were calculated using dfast v1.2.18 [39]. The average nucleotide identity (ANI) and average amino acid identity (AAI) values between the reconstructed and reference CPR genomes were calculated using pyani v0.2.12 and CompareM v0.1.2 (https://github.com/dparks1134/CompareM), respectively [40]. Open reading frames (ORFs) of all genomes were identified using Prodigal v2.6.3 with the “-p single” parameters [41, 42]. For the 12 CPR genomes, annotation of the predicted ORFs was conducted using KofamKOALA with the KEGG database to reconstruct metabolic pathways [43, 44]. The biogeochemical cycling potential of all MAGs was inferred using METABOLIC-G v4.0 [45]. All the available genomes affiliated with CPR bacteria (*n* = 4581), *Thermodesulfovibrionales* (*n* = 226), and CSP1-3 (*n* = 44) were retrieved from the GTDB database (Release 220) and combined with the MAGs to evaluate the functional profiles of potential folate cycling [46]. ORFs were annotated using eggNOG mapper v2.0 with the eggNOG v5.0 database to obtain the KO hits of KEGG annotations with E-values < 1e − 5 [47, 48].

### Construction of the phylogenetic trees

For the phylogenomic analysis of CPR, representative genomes of all the available CPR lineages described in the GTDB database were downloaded. To build the phylogenomic tree for the specific MAG, the representative genomes of the corresponding phylogenetic groups (e.g., order or family) were retrieved from the database. To create the concatenated gene phylogeny, 120 bacterial marker genes from each MAG and the representative reference genomes were extracted and aligned using GTDB-TK according to the official pipeline [36]. Specifically, the sequence alignment was performed using the “gtdbtk identify” and “gtdbtk align” commands. During the “gtdbtk identify” step, ORFs from all genomes were analyzed to identify 120 bacterial marker genes using HMMER v3.4 [49, 50]. These marker genes were then aligned using hmmalign, and the resulting alignment was processed to mask gaps and uncertainties, ensuring a conserved sequence alignment [50]. Finally, the aligned sequences were used for phylogenetic inference. Phylogenetic trees were constructed using IQ-TREE v2.2.2.6, with the best-fit evolutionary models selected by ModelFinder and 1000 ultrafast bootstraps (-T AUTO -m MFP -bb 1000) [51, 52]. The best-fit evolutionary models were “LG + F + I + R10” for the CPR genomes, “Q.insect + F + I + R9” for *Thermodesulfovibrionales*, and “Q.insect + F + I + R4” for CSP1-3, respectively. The 16S rRNA gene sequences extracted from the reconstructed CPR MAGs were recovered using RNAmmer v1.2 and then queried against the NCBI database using Blastn [53]. The 16S rRNA genes and their top 10 hits were aligned using Muscle v5.1, and alignment gaps were removed using trimAl v1.4.rev22 in “automated” mode [54, 55]. A maximum likelihood tree was then inferred using IQ-TREE with the best-fit evolutionary model “TVMe + I + G4” (-T AUTO -m MFP -bb 1000) [51]. All the trees were displayed and annotated using the online tool iTOL [56].

### Co-occurrence network and iRep calculation

High-quality MAGs recovered from the samples were used to analyze the co-occurrence patterns of CPR bacteria with other community members in the hyperalkaline environment. The relative abundance of MAGs was calculated based on the Transcripts Per Million (TPM) method. Quality-trimmed reads from all samples were mapped to the contigs of each MAG using BWA-MEM v0.7.11 [57]. Read counts were normalized with CoverM v0.6.1 (https://github.com/wwood/CoverM) using genome mode with the parameters “–min-read-percent-identity 0.95 –min-read-aligned-percent 0.75 –trim-min 0.10 –trim-max 0.90 –methods tpm”. Co-occurrence analyses were conducted using SparCC with default parameters based on the abundance matrix of the MAGs [58]. A total of 1000 bootstrap samples were used to estimate stable correlation values from the compositional data. Previous co-occurrence analyses in groundwater samples have shown that CPR members are often positively correlated with each other, suggesting similar ecological preferences rather than physical interactions [25]. Thus, only positive SparCC correlations with *r* > 0.9 and statistical significance (*p* < 0.05) between CPR and non-CPR MAGs were retained as potential CPR-hosts pairs [59]. Additionally, genome coverage of the MAGs was calculated using CoverM with the following arguments “-m mean –min-read-aligned-percent 0 –min-read-percent-identity 0 –min-covered-fraction 0”. These flexible settings were chosen to maximize the inclusivity of reads during the calculation of genome coverage [60]. The linear relationship between the genome coverages of CPR genomes and their predicted auxotrophic hosts was assessed through Pearson correlation, utilizing the “cor.test” function from the “vegan” package in R [61]. Index of replication (iRep) values for the MAGs were calculated using iRep v1.10 [62].

### Structural modeling and heterologous expression of *folA* genes

The protein structure of dihydrofolate reductases (DHFRs), encoded by *folA* genes in the reconstructed CPR MAGs was modeled using AlphaFold2, employing a default MSA pipeline within ColabFold [63, 64]. The resulting structure was analyzed using ChimeraX [65]. Conserved domains of DHFRs were identified using the NCBI CD-search tool [66]. The *folA*-deficient strain *Escherichia coli* MG1655 Δ*folA* (MG1655 Δ*folA*) was developed utilizing the Lambda Red recombination system (Table S2) [28]. The CPR-derived *folA* genes encoding DHFRs were artificially synthesized with codon optimization for expression in *Escherichia coli* and cloned into the pBBR1MCS-2 vector. The recombinant plasmids pBBR1MCS-2-*folA* were then transformed into the MG1655 Δ*folA* mutant. Previous studies have shown that the Δ*folA* strain could grow only when exogenous thymidine was amended [67]. Therefore, the metabolic activity of the strains harboring the recombinant plasmids with the CPR-derived *folA* genes was assessed in Luria–Bertani (LB) medium containing kanamycin (50 µg/mL) but lacking thymidine.

## Results and discussion

### Prevalence of CPR bacteria in the hyperalkaline environment

**Fig. 1:**
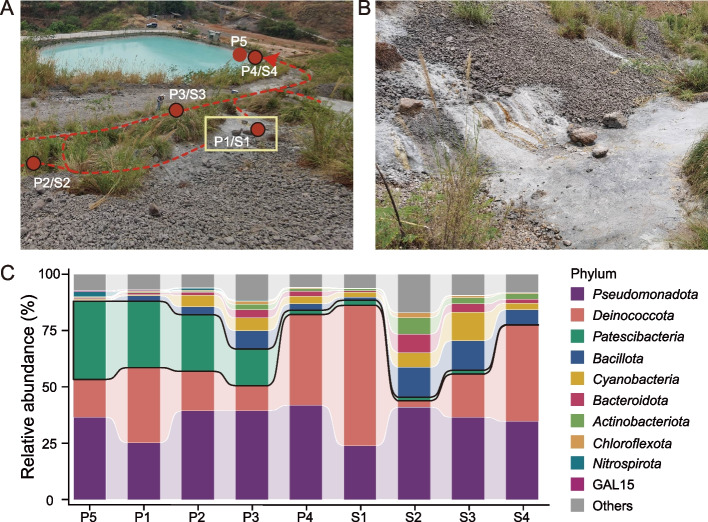
The sampling sites and relative abundance of CPR bacteria in the strongly alkaline environment. **A** Overview of the sampling area in this study. The hyperalkaline leachate flows along the hills with weathering steel slag deposits and is collected in a downstream collection pool. The red circles with black borders represent the leachate samples (P1–P4) and corresponding sediment samples (S1-S4) collected in 2021, while the red circle without the border (P5) indicates the leachate sample collected in 2018. **B** The enlarged view of the yellow-framed area in (A). It illustrates the source of leachate from the steel slag heap and the formation of white carbonate precipitates over the leachate. **C** The microbial community structure was identified using the 16S rRNA genes derived from the raw metagenomic reads and analyzed with phyloFlash [30]. The taxa belonging to CPR (*Ca.* Patescibacteria) were outlined with black solid lines. Only the top 10 taxa by relative abundance were illustrated, with the remaining taxa indicated as “Others”

The leachate with pH > 12 was generated from the hydration of the Ca-rich minerals in the ferrous slags and flowed into the downstream collection pool alongside the slope of the smelting slag deposits (Fig. 1A, B) [27]. Due to the high pH and concentrated Ca^2+^, rapid precipitation of calcium carbonate minerals (e.g., calcite) occurred [27, 68]. The microbial communities in the hyperalkaline environment were examined by analyzing the 16S rRNA gene sequences extracted from the quality-trimmed metagenomic reads (Table S1) [30]. These communities were dominated by *Serpentinimonas* affiliated to phylum *Pseudomonadota* (25.1–41.8%), followed by phylum *Deinococcota* (11.1–40.3%), mainly represented by *Meiothermus* and *Trueperaceae*. Notably, CPR bacteria accounted for 1.93–34.8% of the microbial communities in the oligotrophic and highly alkaline leachate and sediments (Fig. 1C).

### Novel CPR MAGs recovered from ultra-alkaline environment

To further characterize the taxonomy of CPR bacteria retrieved from this highly alkaline environment, MAGs were recovered from metagenomic sequencing data. A total of 12 medium- to high-quality MAGs (bin25-bin36) affiliated with the CPR superphylum were reconstructed from the microbial populations inhabiting the ultrabasic environment (Table S3). These MAGs exhibited reduced genome sizes (0.44–0.70 Mb), with 421 to 714 protein-coding genes and coding densities ranging from 83.2 to 92.4% (Table S3). This suggests that CPR may adapt to nutrient-poor environments through genome streamlining, reducing the costs of some metabolic pathways essential for biosynthesis (e.g., DNA replication and some amino acid synthesis) [25, 69].

**Fig. 2:**
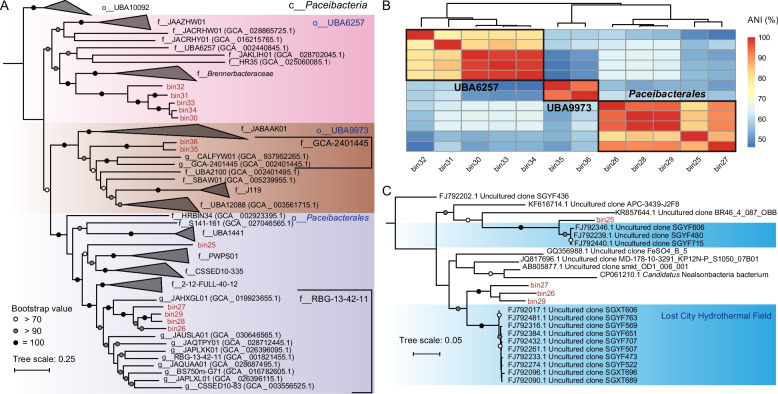
Novel CPR MAGs reconstructed from metagenomic libraries of the samples collected from the ultrabasic environment. **A** The maximum-likelihood phylogenomic tree based on 120 concatenated bacterial marker genes from the CPR MAGs in our study with reference genomes. The CPR MAGs recovered in this study were labeled in red. The CPR genomes affiliated with the orders UBA6257, UBA9973, and *Paceibacterales* were shown in pink, orange, and purple backgrounds, respectively. Ultrafast bootstrap values were represented by colored circles as indicated in the bottom left. **B** Average nucleotide identity of the CPR MAGs in this study. **C** Phylogenetic tree of 16S rRNA genes of the CPR MAGs. The 16S rRNA genes of CPR MAGs identified in this study were labeled in red. Uncultured bacterial clones recovered from the Lost City Hydrothermal Field were shown in blue background and the sequences were retrieved from NCBI

The novelty of these CPR MAGs was confirmed using phylogenetic analyses based on 120 concatenated bacterial marker genes, standardized for genome classification, as used in the GTDB. All the CPR MAGs recovered from the ultra-alkaline environment belonged to class *Paceibacteria* and were affiliated to orders UBA6257, UBA9973, and *Paceibacterales* (Table S3). The 12 recovered CPR MAGs were classified as members of novel species, genera, or even families (Fig. 2A). Specifically, MAGs bin26-bin29 represented the new species within genus JAHXGL01 in family RBG-13–42-11 of *Paceibacterales*. The only species belonging to this genus were previously found in the highly alkaline serpentinized groundwater in The Cedars, CA, USA (Fig. 2A). Suzuki et al. (2024) found that CPR bacterial members accounted for more than 60% of the microbial communities in the deep groundwater of The Cedars [70], similar to the prevalence of CPR members in our samples. MAG bin25 was identified as a member of a new family in the order *Paceibacterales*, while MAGs bin30-bin34 formed a new family closely related to the family *Brennerbacteraceae* of order UBA6257. Additionally, MAGs bin35-bin36 constituted a new genus in the family GCA-2401445, which comprised only two MAGs from a low-oxygen fluid of a marine subsurface aquifer [71]. Among our reconstructed MAGs, only bin26-bin29 and the genus JAHXGL01 exhibited AAI values higher than 60%, while the others were < 60% in AAI with previously published CPR genomes (Table S4). Given that the previously reported genus-level AAI threshold was 60–80%, the AAI analysis confirmed that our detected CPR bacteria were novel taxa [72].

Cluster analysis of ANI revealed a structure similar to that of the phylogenetic analyses, confirming the taxonomic assignments of these CPR MAGs (Fig. 2B). Phylogenetic analysis of the 16S rRNA gene revealed that the close relatives of these CPR MAGs, particularly those belonging to *Paceibacterales*, exhibited high similarity to those reported in the Lost City hydrothermal field, a marine serpentinizing ecosystem (Fig. 2C) [73]. The phylogenetic close relation between the CPR MAGs in these highly alkaline environments (e.g., serpentinites and heavily weathered steel slag leachate) may reflect habitat or ecological niche preferences, or even the availability of hosts for the episymbiotic CPR groups [27, 70, 73].

### Limited metabolic capacities of the CPR MAGs in the ultra-alkaline environment

**Fig. 3:**
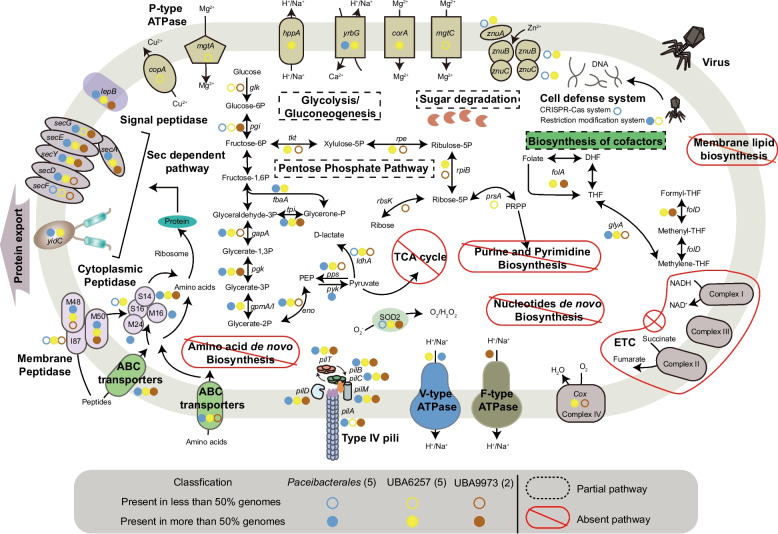
Metabolic reconstruction of the CPR MAGs (*n* = 12) derived from the ultrabasic environment under study. The number of MAGs affiliated with different orders of CPR were indicated as the values in parentheses. Filled circles indicated that the genes were present in > 50% of the MAGs, while the open ones represented the genes present in ≤ 50% of the MAGs. Abbreviations: TCA, tricarboxylic acid cycle; ETC, electron transport chain; PRPP, phosphoribosyl pyrophosphate; PEP, phosphoenolpyruvic acid; SOD, superoxide dismutase. The details of the genes are included in Table S5

Similar to previous studies, our medium- to high-quality CPR MAGs exhibited limited metabolic potential. They showed deficiencies in complete metabolic pathways for purine and pyrimidine biosynthesis, tricarboxylic acid cycle, amino acid biosynthesis, inorganic nitrogen, and sulfur metabolism, and most complexes of the oxidative electron transport chain (Fig. 3 and Table S5) [3]. Unlike the absence of ATPases in the CPR MAGs from marine sediments [10], the genes associated with energy production were widely detected in our CPR MAGs. Specifically, the CPR members of orders *Paceibacteales* and UBA6257 harbored V-type ATPases, while those from order UBA9973 harbored F-type ATPases, suggesting that CPR bacteria in hyperalkaline environment could generate ATP via proton motive force (PMF) [74]. Although the genes encoding hydrogenases that establish PMF were absent, those encoding cytochrome *c* oxidase were found in our CPR genomes (Fig. 3) [75]. This implies that they may possess cytochrome *c* oxidase complex (complex IV) to transfer electrons to oxygen, ultimately producing water and establishing PMF for ATP generation [76]. Thus, the indigenous CPR bacteria may perform respiration in the presence of oxygen, which is different from the previous findings, in which CPR bacteria from anaerobic environments (e.g., groundwater and marine sediments) lacked respiratory electron transport chains and were non-respiring [4, 10]. Furthermore, similar to previously identified CPR bacteria, all our reconstructed CPR genomes contained the genes encoding proteins related to glycolysis and pentose phosphate pathway, suggesting they may synthesize ATP through fermentation and partial glycolysis via substrate-level phosphorylation. This allows for glucose degradation to pyruvate, which can further produce lactate via L-lactate dehydrogenase under microaerobic conditions, reflecting flexible energy generation processes by the CPR bacteria in such a challenging environment (Fig. 3) [4]. Like other reported CPR species, all our CPR genomes also contained nearly complete gene cluster encoding type IV pili and the Sec-dependent protein translocation system [18, 19, 77]. Moreover, the *yrbG* genes, which encode the cation/proton antiporters crucial for pH homeostasis in alkaliphilic prokaryotes, were found in most CPR MAGs affiliated to *Paceibacteales* and UBA6257 (Fig. 3) [27].

Although most pathways for cofactor biosynthesis were absent, a suite of genes (e.g., *folA*, *glyA*, and *folD*) encoding the enzymes responsible for bioactive folate synthesis and cycling was predicted in the CPR MAGs derived from the hyperalkaline environment [78]. These genes encode dihydrofolate reductase, serine hydroxymethyltransferase, and methylenetetrahydrofolate dehydrogenase, respectively, which catalyze the formation of the bioactive forms of folate, including THF and its derivatives (e.g., methylene-, methenyl-, and formyl-THFs) (Fig. 3 and Table S5). In addition, the genes encoding transport proteins (e.g., ATP-binding cassette transporter) were present in some CPR genomes. These results suggest that CPR-guided folate metabolism and exchange may exist in the microbial communities inhabiting the ultrabasic environment.

### Potential folate-mediated CPR-host interactions inferred from community-based analyses

Folate is a vital group of cofactors involved in various cellular processes, including DNA synthesis, repairment, and methylation, as well as amino acid metabolism [79]. Microorganisms depend on folate for nucleotide synthesis, with folate derivatives being essential for the synthesis of purine and thymidylate, the key components of DNA. In amino acid metabolism, folate facilitates the conversion of homocysteine to methionine and the synthesis of serine and glycine [78]. Moreover, microbes that produce folate could support the growth of folate-auxotrophs, promoting cooperative interactions and contributing to the diversity and functionality of microbial communities [80]. Thus, it is reasonable to hypothesize that episymbiotic CPR groups may benefit their hosts through folate metabolism.

**Fig. 4:**
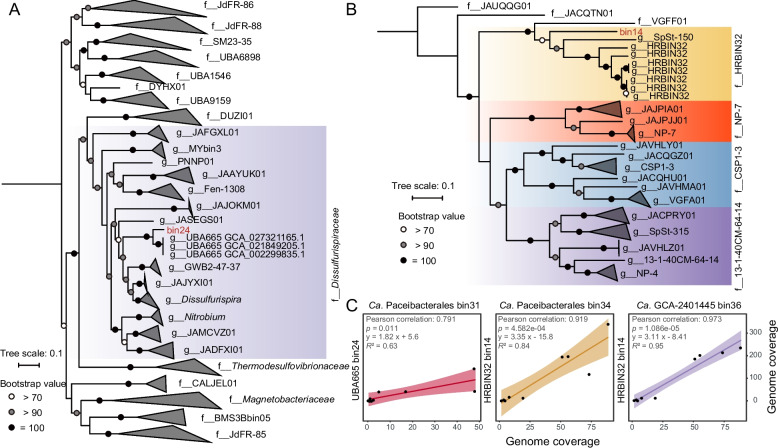
Phylogeny of two potential THF-auxotrophs and their genome coverage correlations with CPR MAGs. **A** The Maximum-likelihood phylogenomic tree of *Ca.* UBA665 bin24 and the reference genomes of order *Thermodesulfovibrionales* from the GTDB database (R220, *n* = 226). The genomes of the family *Dissulfurispiraceae* were highlighted with a purple background. **B** The Maximum-likelihood phylogenomic tree of *Ca.* HRBIN32 bin14 with all the genomes affiliated to phylum CSP1-3 from the GTDB database (R220, *n* = 44). The genomes of different families were shown with the background in different colors. **C** The linear relationship of genome coverages between three CPR genomes and their predicted hosts with significant Pearson correlation

SparCC co-occurrence network analysis implied a potential syntrophic relationship between some CPR and the bacteria affiliated to phyla *Bacteroidota*, CSP1-3, *Deinococcota*, *Bacillota*, and *Nitrospirota* (*r* > 0.9, *p* < 0.05) (Tables S6 and S7). Specifically, a close association was identified between *Nitrospirota* bin24 and CPR bin31. Moreover, bin14 affiliated to candidate phylum CSP1-3 established a significant connection with CPR bin34 and bin36 (Fig. 4 and Table S6). A previous study based on the analysis of 1445 bacterial genomes has shown that *thyA*, which encodes thymidylate synthase, and *folA* exhibit synteny and co-occurrence patterns [78]. Genomic analysis revealed that *Nitrospirota* bin14 and CSP1-3 bin24 contained the *thyA* genes, but their essential *folA* genes were absent. Given the high quality of the two reconstructed genomes (> 99% in completeness and < 5% in contamination), their absence of *folA* was unlikely due to limited sequencing depth (Table S3). Thus, these two species that lacked the *folA* genes might be the potential THF-auxotrophic hosts for the CPR members. Notably, the *folA* genes were present in the symbiotic CPR partners for the two potential THF-auxotrophs (i.e., bin24 and bin14). Additionally, coverage of the CPR MAGs showed a significant positive linear correlation with these two potential THF-auxotrophs, which supported their putative symbiotic relationship (Fig. 4C).

As the potential host for CPR bin31, the bin24 affiliated to order *Thermodesulfovibrionales* of *Nitrospirota* was a potential sulfate-reducing bacterium (Table S8). Phylogenetically related microorganisms have previously been reported in highly alkaline fluids associated with serpentinization (e.g., Lost City hydrothermal field and Samail Ophiolite in Oman) (Fig. 4A) [73, 81]. Despite the lack of culture-dependent evidence, earlier studies demonstrated that CPR species consistently co-occurred with members of the phylum *Nitrospirota* in groundwater [69, 82–84]. Notably, bin24 carried the genes encoding the key CODH/ACS complex (*cdhCDE*) involved in the Wood-Ljungdahl pathway (WLP) for carbon fixation. As WLP is a folate-dependent process [85], the CPR bin31 may facilitate carbon fixation by its *folA-*lacking host bin24 through folate metabolism, thereby enhancing the stability of microbial communities in highly alkaline environments. Meanwhile, the MAG bin14 was affiliated with candidate phylum CSP1-3, which has been found in various environments, including biofilms from alkaline hot springs, ammonia-oxidizing enrichment cultures, and marine ecosystems (Fig. 4B). Members of this phylum typically exhibited diverse metabolic capabilities, involving carbon, sulfur and nitrogen cycling [86–90].

To evaluate the growth of these MAGs in situ, the index of replication (iRep) was calculated from the metagenomes (Table S9). Both the CPR bacteria (e.g., bins 31, 34, and 36) and their potential THF-auxotrophic hosts (e.g., bin14 and bin24) had iRep values higher than 1.0, suggesting that these CPR strains might actively replicate and co-occur with the THF-auxotrophs in this highly alkaline ecosystem.

**Fig. 5:**
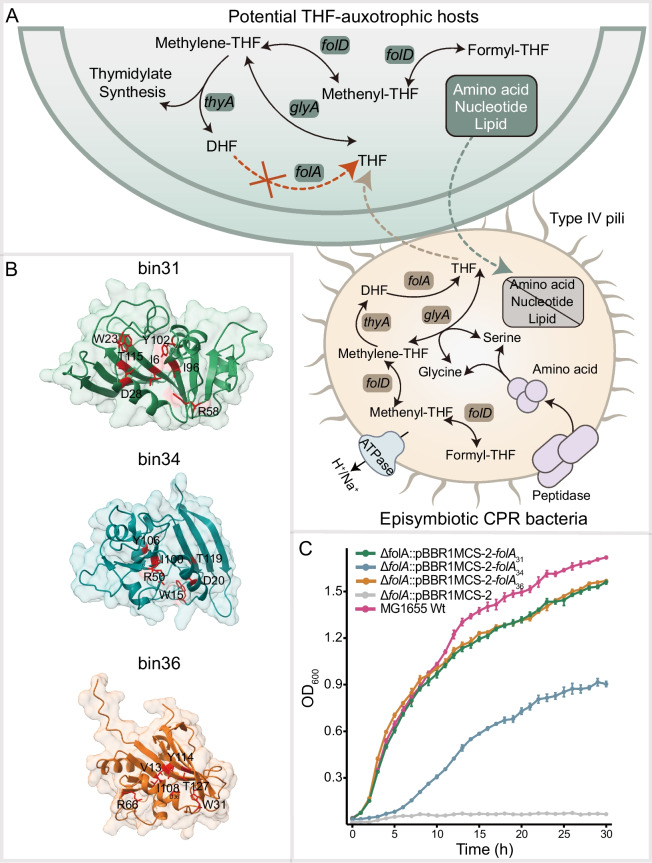
Proposed interaction between our reconstructed CPR species and their potential THF-auxotrophic hosts. **A** The conceptual model infers the interactions between CPR species and their potential THF-auxotrophic microbial hosts. The proposed exchange of THF and other essential cellular components between CPR and their auxotrophic bacterial hosts were shown in dashed lines with arrows. **B** The structural arrangement of the CPR DHFRs encoded by the detected *folA* genes was modeled using Alphafold2 [63]. The folate-binding sites were highlighted in red.** C** The growth curves of the *E. coli* MG1655 wild type, the Δ*folA* mutant containing the empty vector, and the complementary Δ*folA* mutants containing the plasmid pBBR1MCS-2 harboring one of the *folA* genes from CPR bacteria. The error bars indicated the standard deviation of three replicates. Δ*folA*::pBBR1MCS-2: the empty plasmid pBBR1MCS-2 was transformed into strain Δ*folA*; Δ*folA*::pBBR1MCS-2-*folA*_31/34/36_: the recombinant plasmid pBBR1MCS-2 inserted with individual *folA* gene from the CPR MAGs (bin31, bin34 and bin36) was transformed into strain Δ*folA* (Table S2). The complementary strains were incubated in an LB medium without thymidine at 37 °C

In order to assess whether the absence of *folA* for bin14 and bin24 was due to interruption or cutoff in our reconstructed MAGs, the genomic data for all the available *Thermodesulfovibrionales* (*n* = 226) and CSP1-3 genomes (*n* = 44) from GTDB (Release 220) were annotated to investigate the distribution of the *folA* and *thyA* genes (Tables S10–11). The results indicated that 81 *Thermodesulfovibrionales* genomes contained *thyA* genes; but only four or 4.9% of these genomes also retained the *folA* genes (Table S11). These findings suggest that the majority of *Thermodesulfovibrionales* genomes have no *folA* genes and may depend on “public goods” provided by other community members. Among the four *Thermodesulfovibrionales* genomes containing both *folA* and *thyA* genes, the *folA* gene in all of these genomes is not located adjacent to *thyA*, suggesting that in this group of organisms, the *folA* and *thyA* genes may be interrupted. In contrast, among the 19 CSP1-3 genomes with *thyA* genes, 57.9% (*n*= 11) retained the *folA* genes, with no interruptions observed (Table S11). Thus, CSP1-3 members may employ a different strategy, with some capable of synthesizing THF independently, while others may rely on other community members for the essential cofactor. Overall, the high chance of lost *folA* genes in the *Thermodesulfovibrionales*, coupled with the close proximity of *folA* and *thyA* genes in CSP1-3, provide further support for reconstructed *Thermodesulfovibrionales* bin24 and CSP1-3 bin14 as auxotrophs in our studied system (Figs. 5A and S1) [78].

THF, transformed from dihydrofolate by DHFR, functions as a pivotal carrier molecule for various single-carbon moieties (e.g., methyl-, methylene-, methenyl-, and formyl-THFs) and is essential for DNA synthesis and methylation [78, 91]. Notably, the DHFR encoded by *folA* and the thymidylate synthase encoded by *thyA* constitutes a functionally coupled adaptive unit that coordinates maintenance of folate metabolite pools. Thus, deletion of either *folA* or *thyA* can lead to cell death [67, 78]. Almost all the organisms containing *thyA* need *folA* to synthesize thymidylate, an essential DNA precursor [67, 92].

Notably, all our predicted CPR-derived *folA* (CPR-*folA*) genes contained conserved functional domains and essential folate binding sites based on protein structure prediction (Figs. 5B and S2). The enzymatic function of the proteins encoded by the CPR-*folA* genes was further characterized using heterologous expression [78]. The reconstructed plasmids with the CPR-*folA* genes were introduced into a *folA*-deletion mutant of *Escherichia coli* MG1655 (Δ*folA*). Physiological characterization showed that the Δ*folA* was a thymidine-auxotrophic strain and relied on amended thymidine for growth [67]. Upon transformation with the recombinant plasmids inserted with the CPR-*folA* genes, the complementary strains resumed growth without thymidine amendment (Fig. 5C). The successful heterologous expression of the *folA* genes from CPR MAGs suggested that the detected DHFRs of the CPR bacteria were enzymatically functional and could catalyze the biosynthesis of bioactive folate*.* This, combined with the co-occurrence of CPR genomes and their predicted hosts (Table S6), suggests that folate cofactor synthesis by CPR species may facilitate their mutualistic relationship with the hosts [93]. In addition, although co-occurrence networks provide preliminary insights into potential microbial interactions by reflecting spatio-temporal associations in species abundance, the observed positive correlations may also result from shared ecological preferences for similar environmental conditions [25, 84]. The relevance of non-specific interactions within a complex community should also be considered.

Based on the metabolic features of “*Ca*. Paceibacterales” and their THF-auxotrophic hosts, a potential interaction scenario was proposed (Fig. 5A). The THF-auxotrophic hosts may provide amino acids, nucleotides, membrane lipids, and other nutrients for *Ca*. Paceibacterales. In turn, *Ca*. Paceibacterales may support the growth of their auxotrophic hosts by providing essential cofactors. CPR-host interactions may be achieved via pili-like structures based on previously published studies [4, 18, 94]. Pili-associated genes were widely detected in CPR genomes, including those reconstructed in this study (Fig. 3) [12]. Notably, homologs of folate transporters (e.g., *ecfA*) were identified in the host genomes (e.g., *Thermodesulfovibrionales* bin24 and CSP1-3 bin14), while no such proteins were detected in CPR genomes (e.g., bin31, bin34 and bin36) (Table S12). Additionally, both CPR and their host genomes harbor uncharacterized transporters from the ATP-binding cassette family and major facilitator superfamily, which may be involved in folate transport [95, 96].

### Folate cofactors synthesis is prevalent in diverse CPR bacteria

**Fig. 6:**
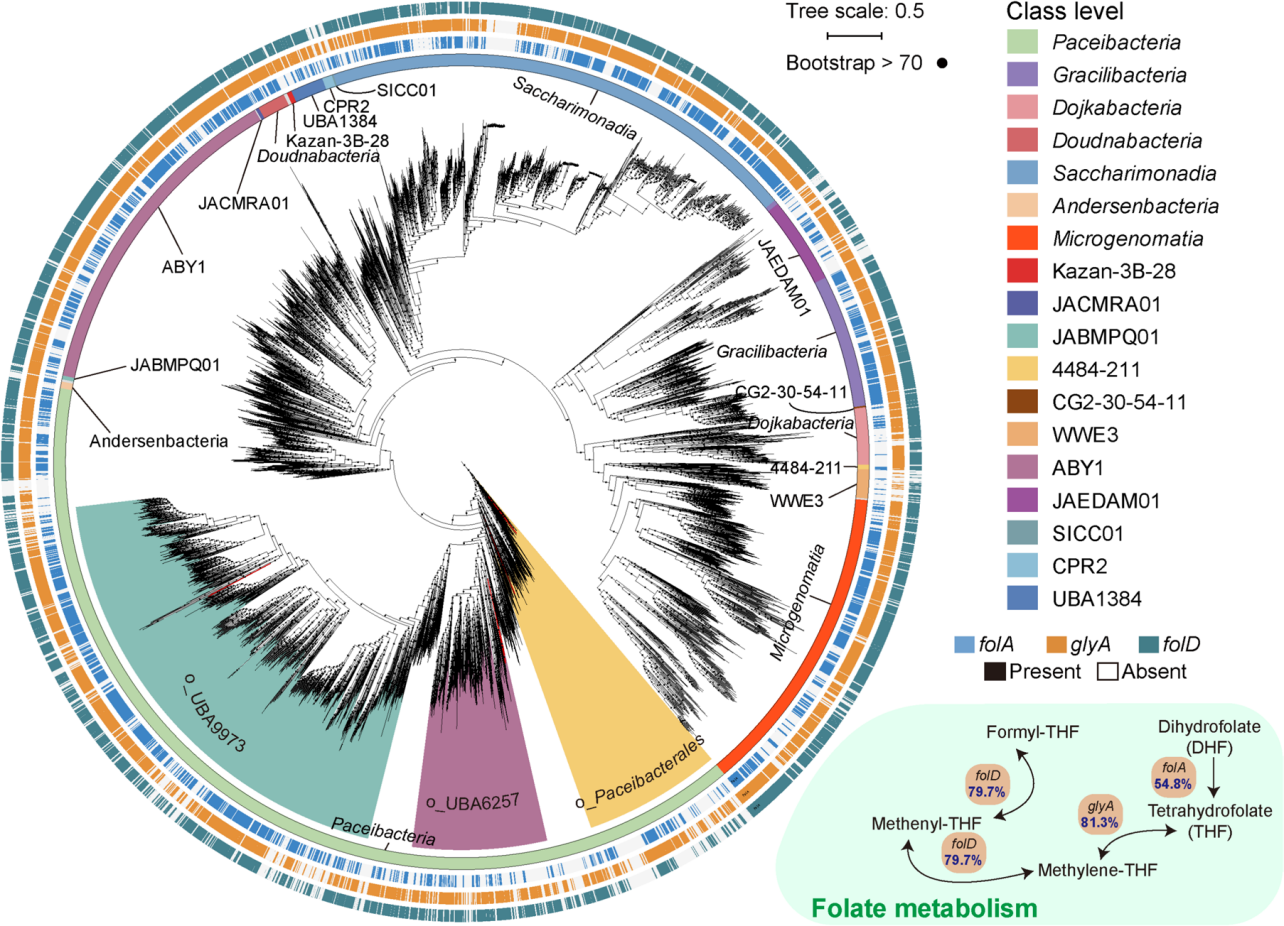
Prevalence of the genes associated with folate cofactor metabolism in the CPR superphylum. The maximum-likelihood phylogenomic tree of CPR from this study (*n* = 12) and the GTDB database (R220, *n* = 4581) was constructed based on 120 concatenated bacterial marker genes. Each class of CPR was represented by distinct colors in the inner circle. The phylogenomic groups comprising the MAGs affiliated with orders *Paceibacterales*, UBA6257, and UBA9973 in the present study were filled with yellow, purple, and blue backgrounds, respectively. From the outermost to the inner circles, the detection of *folD*, *glyA*, *folA*, and the taxonomic affiliation of the CPR bacteria was represented with different colors. The pathways for folate metabolism were illustrated in the bottom right with a green background, showing the proportion of CPR genomes carrying the genes involved in the metabolism of crucial folate cofactors

Although previous studies have demonstrated that CPR generally possesses limited biosynthetic capacity for cofactors, folate biosynthetic capacity has been implied for some members of *Gracilibacteria* in freshwater environments [4, 46]. To determine whether the presence of the genes associated with folate cofactors was a conserved feature in CPR, a collection of available 4581 CPR genomes derived from a variety of ecosystems was retrieved from GTDB (R220) and analyzed. The *folA*, *glyA*, and *folD* genes critical for forming bioactive cofactors were detected in 54.8, 81.3, and 79.7% of the surveyed CPR genomes, respectively (Fig. 6 and Table S13). Intriguingly, 90.8% of the CPR genomes contained at least one of the three genes; about 79.6% and 45.4% of the CPR genomes contained at least two and all of these three genes, respectively (Table S13). These observations suggest that the potential to synthesize bioactive folate cofactors is common for CPR bacteria.

In microbial communities, folate may be crucial not only for the survival of individual organisms but also serve as “public goods” benefiting the entire microbial consortia by promoting nutrient sharing, supporting syntrophic relationships, and enhancing environmental adaptation by microorganisms. The syntrophic metabolism of such “public goods” is prevalent in microbial communities for recycling essential nutrients in many ecosystems [97]. For example, folate cross-feeding between microorganisms has been demonstrated in termite hindguts. Specifically, *Treponema primitia* relied on the essential growth factor, folate, provided by other gut microbiota to support its growth [98, 99]. Folate exchange may be important in oligotrophic environments, where the native microorganisms need to efficiently utilize limited nutrients for biosynthesis and growth. The availability of folate within a microbial community can enhance the adaptive capacity of the ecosystem, enabling microorganisms to thrive in various environmental conditions [100]. The conserved metabolic potential for folate cofactor biosynthesis in CPR bacteria, as implied in this study, underscores their importance in collaboration with and contribution to the coexisting microbial populations [46]. Although the low biomass and highly alkaline condition create challenges for visualization, targeted imaging (e.g., fluorescence in situ hybridization) of CPR bacteria and their potential auxotrophic hosts will significantly enhance the understanding of their association [46]. High-resolution imaging techniques (e.g., transmission electron microscopy) can further elucidate the direct physical interactions between CPR bacteria and their hosts. Moreover, cultivation-based studies are needed to verify whether pili-aided metabolite exchange occurs in CPR-host interactions.

CPR groups rely on other organisms, and the molecules involved in their intercellular interactions may offer valuable biotechnological applications. Recently, Burstein et al. identified a novel CRISPR-Cas system (CasY) in CPR genomes, which presents significant implications for genome editing [101]. Additionally, CPR-C4 proteins, found in thermophilic CPR bacteria, have been characterized to share similarities with human vasohibins, implying the potential in biopharmaceuticals [102]. CPR bacteria were also found in human-associated niches (e.g., oral cavity, gastrointestinal tract, and blood), where they were linked to various diseases. For example, an increase in *Saccharimonadia* was observed in the human oral cavity with periodontitis [103]. The *folA* gene was widely used as a target for compounds that inhibit cell proliferation [91]. Given the prevalence of *folA* genes in CPR genomes and their potential benefit on the hosts, manipulation of *folA* or potentially other genes associated to essential cofactors may pose broad applications in the treatment of pathogens or harmful cells.

## Conclusions

In this study, the prevalence of CPR bacteria in the strongly alkaline environment offers a unique opportunity to investigate this underexplored group of organisms and their potential novel symbiotic strategies with their hosts. To date, only members of the *Saccharimonadia* and *Absconditabacterales* within the CPR lineages have been successfully cultured, allowing characterization of their biology and symbiotic relationships with their hosts [17, 19]. Although stable CPR cultures have not been obtained, our parallel evidence implies that in the oligotrophic, extremely alkaline habitat, the lifestyle of the novel CPR bacteria may be beyond parasitism. Specifically, the CPR bacteria harboring *folA* genes may exhibit symbiotic relationships with *folA*-deficient strains, potentially establishing mutualistic interactions through cross-species folate cycling. DHFR, the key enzymes in this process, have been experimentally validated, providing solid evidence for the CPR bacteria to synthesize bioactive essential cofactors (e.g., folate) [16, 104].

Synergistic metabolic interactions are common in diverse natural microbial communities, often resulting in obligate metabolic dependencies among community members [97]. As one of the oldest bacterial phyla, CPR bacteria are ubiquitous in these environments with active microbial exchange (e.g., soil, groundwater, hypersaline lakes, acid mine drainage, and marine), and host-associated ecosystems (e.g., human oral and gut) [103]. The potential association between CPR bacteria and the THF-auxotrophic, carbon-fixing *Thermodesulfovibrionia* in the ultrabasic environment suggests that CPR bacteria may support cellular growth of their hosts, and thus indirectly facilitate nutrient cycling (Table S8). Considering the fact that essential cofactors are among the metabolites for cross-feeding syntrophic organisms, CPR-derived cofactors may be an optional source for the auxotrophs [105]. Thus, CPR bacteria may play a significant ecological role in a variety of environments supplying folate or other essential cofactors for their peers, thereby contributing to the overall stability, functionality, and resilience of the native microbial communities.

Our integrative methodologies can be instructive for characterizing other enzymatic functions for CPR to verify their critical ecological roles in microbial communities and contributions to biogeochemical processes across various habitats. Additional investigations, including cultivation, microscopy, multi-omic techniques, genetic, biochemical, and metabolic characterization, are underway and will help enhance the understanding of the folate- or other essential cofactor-dependent symbiotic relationships between CPR bacteria and their hosts. Recent advancements in techniques, such as EpicPCR (emulsion, paired isolation, and concatenation PCR) and high-throughput cultivation, have led to significant breakthroughs in CPR-specific enrichment [18, 83], which may provide valuable guidance for the further isolation of these enigmatic bacteria.

## Supplementary Information

Additional file 1 : Figure S1. In the genomic context of two THF-auxotrophs (MAGs bin14 and bin24), no *folA* (K00287) was detected close to the gene *thyA* (K00560), while both *folA* and* thyA* genes were detected in the CPR genomes (i.e., MAGs bin31, bin34, and bin36). The *folA* and *thyA* genes were highlighted in yellow. The genes annotated using the KEGG database with KO numbers were highlighted in grey, while those without annotations were shown in white. The detailed information about the genomic context of the scaffold harboring *folA* or *thyA* of CPR bacteria and their potential THF-auxotrophic hosts was provided in Table S10. Figure S2. Amino acid sequence alignment of DHFRs. The conserved folate and NADP^+^ binding sites were highlighted with red and blue background, respectively.

Additional file 2 : Table S1. Sampling and sequencing information for metagenomic data used in this study, including geographic locations, sequencing platform, data size, NCBI accession numbers, and source references. Table S2. List of bacterial strains and plasmids used in this study. Table S3. Characteristics for 38 MAGs, including quality, taxonomic assignment based on GTDB taxonomy. Table S4. Average amino acid identity (AAI) between our reconstructed CPR MAGs and reference CPR genomes from GTDB. Table S5. List of genes assigned to metabolic features of the 12 CPR MAGs. Table S6. Co-occurrence network showed that positive connections between CPR and non-CPR MAGs detected in this study. Table S7. Relative abundance (expressed as TPM values) and genome coverage for the hyperalkaline MAGs in each metagenome. Table S8. Presence and absence of the genes associated with element cycling (e.g., C, N, S, and O). Table S9. The index of replication (iRep) values for the MAGs reconstructed in the metagenomes. Table S10. Immediate genomic context for the scaffold harboring *folA* or *thyA* of the CPR bacteria and their potential THF-auxotrophic hosts. Table S11. The distribution of the *folA* and *thyA* genes in all *Thermodesulfovibrionales* (*n* = 226) and CSP1-3 (*n* = 44) genomes from GTDB (Release 220). Table S12. Potential transporters of folate molecules in the genomes of CPR and their auxotrophic hosts. Table S13. Classification and metabolic potential of folate cofactors of CPR genomes from this study and GTDB database.

## Data Availability

The metagenomic reads are publicly available at the National Omics Data Encyclopedia (NODE) under Project IDs OEP003545 and OEP004270, and the NCBI under Project IDs PRJNA997081 and PRJNA861894. Sequences of CPR and the non-CPR MAGs can be found on figshare (10.6084/m9.figshare.26510536). The accession numbers for the representative CPR genomes from the GTDB database (R220) were listed in Table S13.
