# Supplementary material for: Candidate Phyla Radiation (CPR) bacteria from hyperalkaline ecosystems provide novel insight into their symbiotic lifestyle and ecological implications
Source: Microbiome. 2025 Apr 7;13:94. doi: 10.1186/s40168-025-02077-y (PMC11974145; doi:10.1186/s40168-025-02077-y)
Supplement: Supplementary file 2 — Additional file 1 : Figure S1. In the genomic context of two THF-auxotrophs (MAGs bin14 and bin24), no folA (K00287) was detected close to the gene thyA (K00560), while both folA and thyA genes were detected in the CPR genomes (i.e., MAGs bin31, bin34, and bin36). The folA and thyA genes were highlighted in yellow. The genes annotated using the KEGG database with KO numbers were highlighted in grey, while those without annotations were shown in white. The detailed information about the genomic context of the scaffold harboring folA or thyA of CPR bacteria and their potential THF-auxotrophic hosts was provided in Table S10. Figure S2. Amino acid sequence alignment of DHFRs. The conserved folate and NADP+ binding sites were highlighted with red and blue background, respectively. [file 40168_2025_2077_MOESM1_ESM.docx]

**Supplementary Information for**

**Candidate Phyla Radiation (CPR) from hyperalkaline ecosystems provide novel insight into their symbiotic lifestyle and ecological implications**

Yu He^1^, Shiyan Zhuo^1^, Meng Li^2^, Jie Pan^2^, Yongguang Jiang^1^, Yidan Hu^1^, Robert A. Sanford^3^, Qin Lin^4^, Weimin Sun^5^, Na Wei^6^, Shuming Peng^7^, Zhou Jiang^1^, Shuyi Li^1^, Yongzhe Li^1, 8^, Yiran Dong^1, 9, 10, 11^*, Liang Shi^1, 9, 10^

^1^ School of Environmental Studies, China University of Geosciences, China

^2^ Archaeal Biology Centre, Synthetic Biology Research Center, Shenzhen Key Laboratory of Marine Microbiome Engineering, Key Laboratory of Marine Microbiome Engineering of Guangdong Higher Education Institutes, Institute for Advanced Study, Shenzhen University, Shenzhen, China

^3^ Department of Earth Science & Environmental Change, University of Illinois Urbana-Champaign, U. S.

^4^ Shanghai Biozeron Biological Technology Co. Ltd, China

^5^ Guangdong Institute of Eco-environmental and Soil Science, Guangdong, China

^6^ Department of Civil and Environmental Engineering, University of Illinois Urbana-Champaign, U. S.

^7^ Institute of Ecological Environment, Chengdu University of Technology, China

^8^ Central & South China Municipal Engineering Design and Research Institute Co, Ltd., Wuhan, China

^9^ State Key Laboratory of Biogeology and Environmental Geology, China University of Geosciences (Wuhan), China

^10^ State Environmental Protection Key Laboratory of Source Apportionment and Control of Aquatic Pollution, Ministry of Ecology and Environment, China

^11^ Hubei Key Laboratory of Yangtze Catchment Environmental Aquatic Science, China

* Corresponding author. Mailing address: 388 Lumo Road, Wuhan, China, 430074. Phone: +11(27)67883152. Email: dongyr@cug.edu.cn

**SUPPLEMENTARY TABLES**

**Please refer to Excel files for supplementary tables.**

**Table S1.** Sampling and sequencing information for metagenomic data used in this study, including geographic locations, sequencing platform, data size, NCBI accession numbers, and source references.

**Table S2.** List of bacterial strains and plasmids used in this study.

**Table S3.** Characteristics for 38 MAGs, including quality, taxonomic assignment based on GTDB taxonomy.

**Table S4.** Average amino acid identity (AAI) between our reconstructed CPR MAGs and reference CPR genomes from GTDB.

**Table S5.** List of genes assigned to metabolic features of the 12 CPR MAGs.

**Table S6.** Co-occurrence network showed that positive connections between CPR and non-CPR MAGs detected in this study.

**Table S7.** Relative abundance (expressed as TPM values) and genome coverage for the hyperalkaline MAGs in each metagenome.

**Table S8.** Presence and absence of the genes associated with element cycling (e.g., C, N, S, and O).

**Table S9.** The index of replication (iRep) values for the MAGs reconstructed in the metagenomes.

**Table S10.** Immediate genomic context for the scaffold harboring *folA* or *thyA* of the CPR bacteria and their potential THF-auxotrophic hosts.

**Table S11.** The distribution of the *folA* and *thyA* genes in all *Thermodesulfovibrionales* (N = 226) and CSP1-3 (N = 44) genomes from GTDB (Release 220).

**Table S12.** Potential transporters of folate molecules in the genomes of CPR and their auxotrophic hosts.

**Table S13.** Classification and metabolic potential of folate cofactors of CPR genomes from this study and GTDB database.

**SUPPLEMENTARY FIGURES**


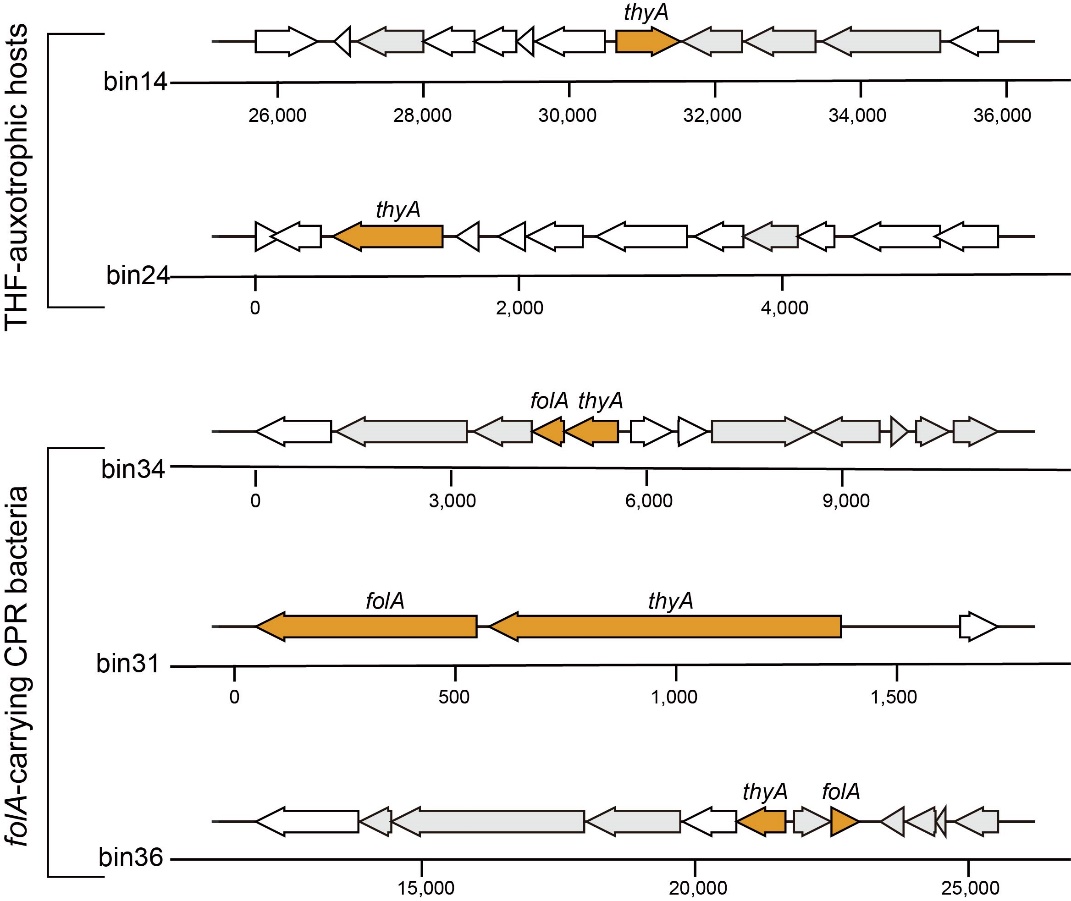


**Fig. S1** In the genomic context of two THF-auxotrophs (MAGs bin14 and bin24), no *folA* (K00287) was detected close to the gene *thyA* (K00560), while both *folA* and *thyA* genes were detected in the CPR genomes (i.e., MAGs bin31, bin34, and bin36). The *folA* and *thyA* genes were highlighted in yellow. The genes annotated using the KEGG database with KO numbers were highlighted in grey, while those without annotations were shown in white. The detailed information about the genomic context of the scaffold harboring *folA* or *thyA* of CPR bacteria and their potential THF-auxotrophic hosts was provided in Table S10.


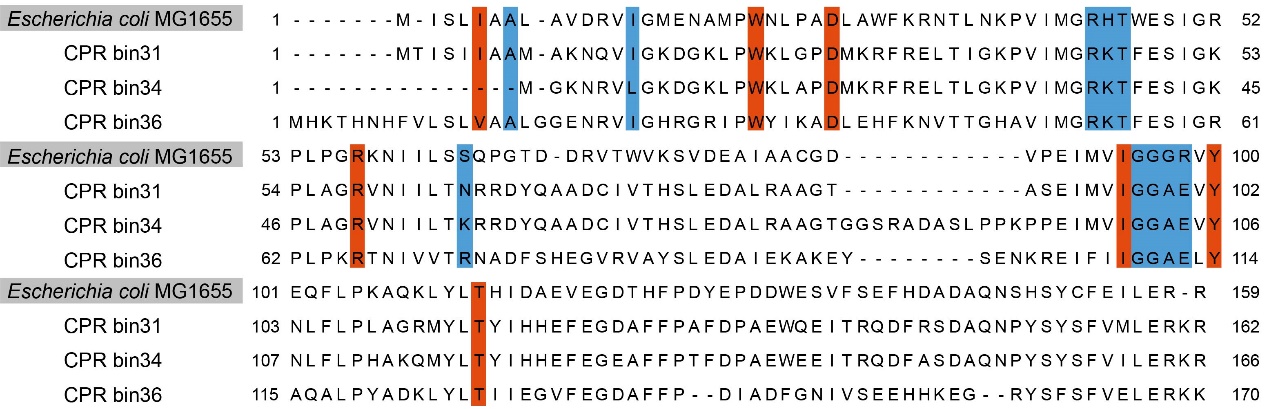


**Fig. S2** Amino acid sequence alignment of DHFRs. The conserved folate and NADP^+^ binding sites were highlighted with red and blue background, respectively.
